# The Coevolution of Fungal Mitochondrial Introns and Their Homing Endonucleases (GIY-YIG and LAGLIDADG)

**DOI:** 10.1093/gbe/evaa126

**Published:** 2020-06-25

**Authors:** Amalia H Megarioti, Vassili N Kouvelis

**Affiliations:** Department of Genetics and Biotechnology, Faculty of Biology, National and Kapodistrian University of Athens, Greece

**Keywords:** fungi, mitochondrial genomes, introns, LAGLIDADG, GIY-YIG, evolution

## Abstract

Fungal mitochondrial (mt) genomes exhibit great diversity in size which is partially attributed to their variable intergenic regions and most importantly to the inclusion of introns within their genes. These introns belong to group I or II, and both of them are self-splicing. The majority of them carry genes encoding homing endonucleases, either LAGLIDADG or GIY-YIG. In this study, it was found that these intronic homing endonucleases genes (HEGs) may originate from mt free-standing open reading frames which can be found nowadays in species belonging to Early Diverging Fungi as “living fossils.” A total of 487 introns carrying HEGs which were located in the publicly available mt genomes of representative species belonging to orders from all fungal phyla was analyzed. Their distribution in the mt genes, their insertion target sequence, and the phylogenetic analyses of the HEGs showed that these introns along with their HEGs form a composite structure in which both selfish elements coevolved. The invasion of the ancestral free-standing HEGs in the introns occurred through a perpetual mechanism, called in this study as “aenaon” hypothesis. It is based on recombination, transpositions, and horizontal gene transfer events throughout evolution. HEGs phylogenetically clustered primarily according to their intron hosts and secondarily to the mt genes carrying the introns and their HEGs. The evolutionary models created revealed an “intron-early” evolution which was enriched by “intron-late” events through many different independent recombinational events which resulted from both vertical and horizontal gene transfers.

**Significance**

Fungal mitochondrial (mt) genomes are abundant with group I and II introns, which usually carry genes (homing endonucleases genes [HEGs]) encoding homing endonucleases. These homing endonucleases play important role in mt intron splicing. Until recently, the evolution of these elements was never examined in a pan genomic level but only in certain mt genes. In this study, the coevolution of these composite elements is revealed, through phylogenetic approaches, analyses of their insertion target sites, the intronic secondary structure changes, and their distribution to the mt host genes. The fungal ancestor most probably had introns and HEGs independent, but HEGs and their introns coevolved through recombination, transposition, and horizontal gene transfer, as presented in our proposed “aenaon” model.

## Introduction

Mitochondria are semiautonomous organelles responsible mainly for cellular respiration and they contain their own genomes (Burger et al. 2003). In fungi, mitochondrial DNA (mtDNA) is found in either circular or linear form, in many copies within the same cell and their size is considerably smaller than the nuclear genome but highly variable (Burger et al. 2003; Aguileta et al. 2014). Their diversity is a product of variation in genomes’ sizes and synteny (Kouvelis et al. 2004; Pantou et al. 2008; Formey et al. 2012). More specifically, their size can vary from 11 kb in *Hanseniaspora uvarum* (Pramateftaki et al. 2006) to 230 kb in *Morchella importuna* (Liu et al. 2020). This divergence is mainly due to the variability in size of intergenic regions and number of introns and intronic open reading frames (ORFs) (Bullerwell et al. 2003; Sethuraman et al. 2009; Joardar et al. 2012; Deng et al. 2018).

Almost all introns found in fungal mt genomes can be classified into two main categories: group I and group II. Both types minimize their effect in the host genome by self-catalyzation including two transesterification reactions by which a mature mRNA is produced, but still they present several differences within their mechanisms of catalysis (Michel and Westhof 1990; Hausner et al. 2014; Zhao and Pyle 2017). The main difference concerning their mechanisms of splicing is that group I introns initiate their catalysis by nucleophilic attack of a guanosine at the 5′ end of their splice site and only after excision, group I introns may circularize, contributing to a shift of the equilibrium toward the spliced products. On the other hand, group II introns initiate their splicing with the formation of a lariat. This is formed when the 5′ end of the intron is linked by phosphodiester bond to an adenosine found near the 3′ end of the intron, similarly to the nuclear mRNA introns which splice via a spliceosome. As for their structural differences, group II introns are structured as six helical domains (I–VI) radiating from a central wheel (named J) or capped by loops (named L), whereas group I introns share a series of short conserved sequence elements (named P–S) and consist of a few hairpins, denoted P1–P10. In detail, group I introns of subtype B contain the minimum conserved helices, as helices P2, P2.1, P5(a–c), P6b, and P9(a, b, 9.1, 9.2) are missing. The second less compact is IA, which has the same structure as IB plus P6b hairpin and subtype ID follows with the addition of P2. IE is the second most hairpin rich subtype containing all helices with the exception of P5 (a–c). Subtype IC is the most expanded one lacking only P6b helix (Saldanha et al. 1993).

Self-splicing introns often include ORFs of homing endonucleases (HEs) (Belfort and Roberts 1997; Chevalier and Stoddard 2001). HEs are enzymes that recognize site-specific DNA targets and are mostly encoded by genes (homing endonucleases genes [HEGs]) in group I introns (Belfort and Roberts 1997; Chevalier and Stoddard 2001). The HEG could be an independent ORF in the intron or in fusion with the upstream exon and get removed post-translationally via proteolysis (Guo et al. 1995). HEGs may also be found as free-standing ORFs or rarely in group II introns (Toor and Zimmerly 2002). ORFs are mainly positioned in loops of the intron that do not interfere with its splicing efficiency. It is proposed that, introns with HEGs invade all the intron-free alleles, through mechanisms, like double-strand breaks or single-stranded nicks and homologous recombination (Belfort et al. 2002). This process is known as “homing” (Burt and Koufopanou 2004). Some HEGs move independently through a double-strand break repair mechanism from an “ORF-containing” intron to an “ORF-less” intron (Sellem and Belcour 1997). Subsequently, HEGs accumulate mutations, get degenerated, and lead to the loss of the intron. It is also proposed that those mutations in the HEGs lead to novel target recognition sequences, allowing them to enter novel sites and promoting the transposition of their intronic host (Mullineux et al. 2011). This is the commonly known life cycle of introns (Goddard and Burt 1999; Gogarten and Hilario 2006). There are four families of HEs, based on conserved aminoacid motifs that participate in the active site of the enzyme: the GIY-YIG, LAGLIDADG, His-Cys box, and HNH (Stoddard 2014). Only genes coding GIY-YIG (GIY) and LAGLIDADG (LD) endonucleases can be found in the fungal mtDNAs (Belfort et al. 2002).

LD endonucleases constitute the most widespread family of HEs in organelles and microbial genomes. The wide proliferation of LDs is due to their ability to recognize a variety of target sequences including group I and group II introns, Archaeal introns, and inteins (Belfort and Roberts 1997; Chevalier and Stoddard 2001; Toor and Zimmerly 2002). LD proteins include one or two copies of the conserved motif. It is suggested that the two motif LDs are produced by duplication and fusion of the motif (Lucas et al. 2001; Haugen and Bhattacharya 2004). The single motif LDs are homodimerized recognizing mostly palindromic repeats in the DNA target, whereas double motif LDs are active monomers and are not restricted to palindromic DNA target sequences (Gimble 2000; Lucas et al. 2001). LDs recognize 18–22 bp target sequences, with recognition flexibility (Chevalier et al. 2005).

GIY endonucleases comprise the second most abundant family of HEs and they are characterized by a conserved aminoacid motif GIY-(X_10–11_)-YIG. This motif has been detected in some repair systems and in restriction enzymes (Kowalski et al*.* 1999). GIY have been found as free-standing ORFs as well as within transposable group I introns in the fungal mtDNAs, in algae and in the cpDNA of plants (Bell-Pedersen et al. 1990; Stoddard 2005). In a single case, an ORF of GIY endonuclease has been found within a group II intron (Lang et al. 2002). Biochemical analyses have shown that GIY endonucleases act as monomers and contain up to five distinct aminoacid motifs.

Phylogenetic and distribution studies of introns and HEGs in fungal mtDNAs were mostly restricted to species of the same genus (*Ceratocystis* species: Sethuraman et al. 2013) or same family (Saccharomycetaceae in Goddard and Burt 1999). In a few studies only, certain mt genes like *rns* and *rnl* (Toor and Zimmerly 2002), *rns* (Mullineux et al. 2011), *cob* (Guha et al. 2018), and *nad*5 (Zubaer et al. 2019) were examined. Moreover, certain LD types, as the LDs of two motifs (Dalgaard et al. 1997), and sequences related to a certain twintronic ORF were analyzed (Guha and Hausner 2014).

The abundance of introns and their HEGs in the fungal mt genomes between the different orders of the fungal subphyla and phyla may be evolutionary explained with two contradicting theories proposed for all organisms: the “Early Intron” versus the “Late Intron” theory. The first theory suggests that introns were abundant in the ancestral genes and a general evolutionary process dominated toward the loss of introns (Goddard and Burt 1999; Gonzalez et al. 1999). The second theory supports intron mobility and thus, expansion within genes due to events of horizontal transfer, even between distant phylogenetically species (Vaughn et al. 1995; Gonzalez et al. 1998). Therefore, the evolution of fungal mt introns and their HEGs is complex and not fully resolved.

Until recently, the known complete fungal mt genomes were few (approx. 160) from certain fungal orders which included species with a medical and/or biotechnological interest (Korovesi et al. 2018). However, whole-genome shotgun (WGS) analyses have resulted in the increasing number of mtDNA sequences (more than double) and the upcoming necessity for correct annotation and characterization of those became apparent, as in the case of *Cryptococcus* (Kortsinoglou et al. 2019), in order to exploit them for typing the fungal species which carry them and additionally decipher the evolution of these genomes.

Therefore, there has never been a pan kingdom synthesis of the evolution of mt introns across genes and taxa, similar to the recent study of the *rps*3 gene (Korovesi et al. 2018). Additionally, this fragmented approach of studying the introns and their HEGs in specific mt genes of fungi confine the analyses only in the phylogenetic relationships of these genes and in restricted evolutionary studies of the fungal mt genomes.

In this work, an effort was made to elucidate the evolution of introns within the mt genes that carry them, based on the analyses of HEGs and their introns from whole fungal mitochondrial genomes.

## Materials and Methods

### Data Retrieval, Annotation, and Characterization of Introns and Their HEs

In this study, mtDNAs of 132 representatives of all known fungal orders containing species with sequenced complete mt genomes were selected. The mt genomes were retrieved from GenBank and 71 of them were annotated or further reannotated for the presence of introns and intronic ORFs (supplementary table 1, Supplementary Material online). The aim was to include in this study, HEs from representatives of almost every known fungal order. Therefore, the matrix of this work contains mt genomes, their introns, and their ORFs from 33 Pezizomycotina, 58 Saccharomycetes (5 major groups), 4 Taphrinomycotina, 17 Basidiomycota, and 20 EDF. The latest group includes the fungal phyla that diverged early during fungal evolution. In detail, this group contains data from representatives which belong to Blastocladiomycota (2 species), Zoopagomycota (2), Chytridiomycota (7), Mucoromycota (7), and Cryptomycota (2) (table 1). All these phyla were analyzed altogether as EDF because they were underrepresented. Furthermore, Saccharomycetes were classified into five main groups: the CTG group (17 species), Pichiaceae (4 species), Phaffomycetaceae (4 species), post-whole-genome duplication (13 species), and pre-whole-genome duplication (20 species) (supplementary table 1, Supplementary Material online).

**Table 1:** Taxonomic Distribution of Representative Fungal Species Whose mt Genomes, Introns, and HEGs Are Examined

Phylum	Subphylum	Order	Number of Strains	Number of Introns with HEGs	Number of GIY-YIGs (f)	Number of LDs (f)
Ascomycota	Pezizomycotina	Capnodiales	1	0	0	0
		Chaetothyriales	1	0	0	0
		Diaporthales	2	14	7	8
		Eurotiales	2	4	0	4
		Glomerellales	3	0	0	0
		Helotiales	3	18	9 (2)	13 (2)
		Hypocreales	3	10	7	3
		Lecanorales	1	0	0	0
		Microascales	1	30	9	22
		Onygenales	4	2	1 (1)	2
		Ophiostomatales	1	0	0	0
		Peltigerales	2	24	7	17
		Pezizales	1	16	4 (2)	27 (12)
		Pleosporales	2	4	2	2
		Sordariales	3	25	9 (1)	20 (3)
		Xylariales	2	50	12	39
		Xylonomycetales	1	1	0	1
	Saccharomycotina	Saccharomycetes	95	92	11	87 (6)
	Taphrinomycotina	Pneumocystidales	1	0	0	0
		Schizosaccharomycetales	2	5	0	5
		Taphrinales	1	7	0	7
Basidiomycota	Agaricomycotina	Agaricales	2	19	3	16
		Cantharellales	1	13	2	11
		Corticiales	1	25	9 (1)	19 (2)
		Polyporales	1	4	2	2
		Sebacinales	1	0	0	0
		Tremellales	2	3	1	2
	Pucciniomycotina	Microbotryales	1	8	2 (2)	8
		Pucciniales	3	6	0	6
		Sporidiobolales	1	7	5	2
	Ustilagomycotina	Tilletiales	1	0	0	0
		Ustilaginales	1	11	2	9
		N/A	1	3	0	3
		Microstomatales	1	0	0	0
Blastocladiomycota	N/A	Blastocladiales	2	3	5 (3)	1
Chytridiomycota	N/A	Chytridiales	4	18	7 (2)	13
		Synchytriales	1	4	0	4
		N/A	1	1	0	1
		Spizellomycetales	1	4	2 (1)	3
Zoopagomycota	Kickxellomycotina	Harpellales	1	13	2	11
	N/A	Entomophthorales	1	21	3	18
Mucoromycota	Mortierellomycotina	Mortierellales	1	3	5 (3)	2 (1)
	Mucoromycotina	Mucorales	2	14	10 (4)	8
	Glomeromycotina	Glomerales	2	6	6 (6)	6
	N/A	Diversisporales	2	6	0	8 (2)
Cryptomycota	N/A	N/A	2	1	0	1

Note.—Numbers in parentheses (f) indicate free-standing HEGs.

Every intron containing an intronic ORF which encoded for either GIY or LD endonuclease was categorized into the main intron groups and subgroups. This characterization was accomplished using the ERPIN algorithm (Eddy and Durbin 1994) of the RNAweasel (Lang et al. 2007) and the “Comparative RNA Web Site and Project” (RNAcentral Consortium 2019). In total, introns that included HEGs were characterized and classified into the main intron categories (IA, IB, IC, ID, and group II introns) by detecting conserved secondary structure elements and the sequence of the P7:P7′ pair. The intronic loops that host HEGs were determined using previous studies (Michel and Westhof 1990; Cech et al. 1994; Hausner et al. 2014) and the Comparative RNA Web Project (Gardner et al. 2011). In each intron, the P7:P7′ pair sequence was detected, if possible, and by using the model structures from representative species like *Tetrahymena thermophila* (Kan and Gall 1982), the loop with the ORF was retrieved. Moreover, all the GIY and LD ORFs were identified manually using the BLAST algorithm (BlastX) (Altschul et al. 1990) against ORFs of known HEGs from phylogenetically related species. Free-standing ORFs were spotted using ORFfinder (Sayers et al. 2011) and Lasergene (Seqman) (Burland 2000). In every case, the ORF size, the protein size, and the insertion site of each ORF inside the intron were identified. Some HEGs were characterized as exonic–intronic, meaning that the start codon was detected upstream of the intron’s limits. ORFs outside mt introns were characterized as free standing. Moreover, the insertion sequence of each intron was retrieved in order to detect possible conserved recognition motifs of HEs (supplementary tables 2 and 3, Supplementary Material online). In the case of LDs, their type was additionally identified as LD1, LD1(2m), LD2, and LD3 using BlastX and protein sequence similarities against known, well characterized LDs (supplementary table 3, Supplementary Material online).

### Phylogenetic Analyses

In total, 129 and 339 protein sequences from the complete data set (supplementary tables 2 and 3, Supplementary Material online) composed the matrices for the phylogenetic analyses of GIY and LD endonucleases, respectively (data available upon request). The main criteria for their inclusion in the analysis were the representation of all fungal orders, if possible, and the sizes of the HEs. In detail, HEs with sizes ranging below 120 aa and above 500 aa were excluded, because most probably the smaller HEs are not functional and the largest may be the result of a gene-fusion or wrong annotation. In the case of the class of Saccharomycetes, the selection was further based on the variability of LD types and introns, due to the extremely large plethora of data within this class (supplementary table 3, Supplementary Material online). Thus, in the matrices, at least one representative from every Saccharomycetes group was kept (see above). Independent Neighbor-Joining (NJ)-based analyses of LD and GIY phylogenies were performed additionally, in order to verify if the conclusions of the detailed phylogeny are applied also in the five different phyla of EDF, even if few data are available. Overall, the phylogenetic analyses for GIY endonucleases include 129 protein sequences from 25 orders, that is, 10, 7, 8, and 7 from Pezizomycotina, Saccharomycetes, Basidiomycota, and EDF, respectively. The LD phylogenetic analyses include 339 protein sequences from 31 orders. Those orders belong to Pezizomycotina (13), Saccharomycetes (14), Taphrinomycotina (1), Basidiomycota (9), and EDF (8).

The protein sequences of GIY and LD endonucleases were aligned using the ClustalW algorithm (Thompson et al. 1994) in Megalign of Lasergene (Burland 2000) with default parameters and got manually edited using PAUP (Swofford 2002). Manual editing was based on the verification and correction of the alignment for the conserved aminoacid motifs of each endonuclease (matrices available upon request). A phylogenetic tree for every HE was produced after employing PAUP for the NJ method and MrBayes (ver. 3) for the Bayesian Inference (BI) (Ronquist and Huelsenbeck 2003). For both methodologies, the parameters used were as described in previous studies (Kouvelis et al. 2004; Korovesi et al. 2018). In all analyses, the respective HEs of *Allomyces macrogynus* were used as outgroup. In addition, the NJ tree was produced with default parameters based on GTR parameters and the support of tree topologies was statistically examined with a bootstrap analysis based on 10,000 replicates in both matrices, that is, the GIY and LD matrices. Moreover, for the BI analyses, the ProtTest program (ver. 1.3) (Abascal et al. 2005) was used in order to define the best fitted evolutionary model for the data sets of HEs. For the GIY data set, the best fitted model was proved to be the WAG + G with *a* = 2.60 as determined after employing the Akaike Information Criterion and Bayesian Information Criterion. Similarly, the most appropriate model for the LD data set, turned out to be the WAG + G with *a* = 2.07, too. In both data sets (i.e., GIY and LD matrices), four independent MCMCMC searches were performed. For each data set, different random starting points were used and after setting the number of generations to 40M and 10M and sampling every 4,000 and 1,000 generations for the LD and GIY, respectively. The burn-in was set to 40,000 and 10,000. Convergence was checked visually after plotting likelihood scores versus generation for the four runs, in both cases.

## Results

### Distribution of Introns Containing HEGs

**Fig. 1.**
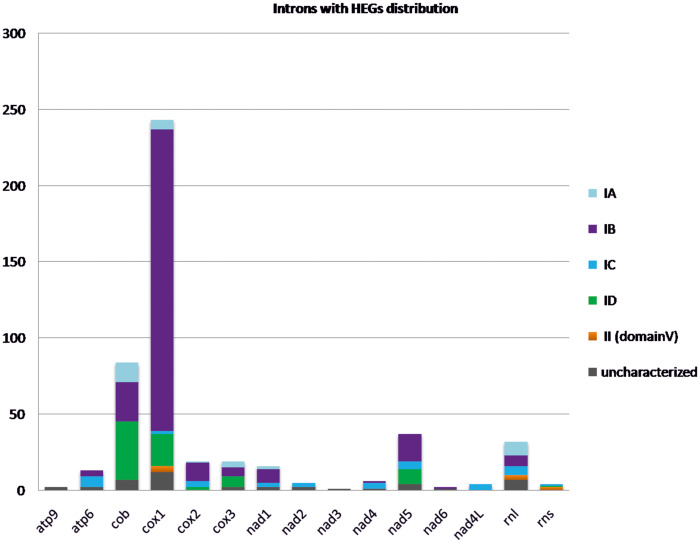
Occurrence of introns hosting either GIY or LD genes (quantified in the *y* axis) at the commonly found mt genes. Different intron subtypes are shown in different colored blocks.

Introns are commonly found in the mt genes which are conserved at the fungal mt genomes. In detail, the genes mentioned are those implicated in ATP production, that is, genes of ATP synthase subunits (*atp*6, *atp*8, and *atp*9), the oxidative phosphorylation, that is, genes of NADH dehydrogenase subunits (*nad*1–6 and *nad*4L), apocytochrome b (*cob*), cytochrome C oxidase subunits (*cox*1–3), and in the mt ribosomes, that is, genes of large and small rRNA subunits (*rnl* and *rns*, respectively). From the 487 identified introns carrying HEGs located in 132 fungal mitochondrial genomes (analyzed in this work), half of the introns (50%) in this study were located in *cox*1 gene and the majority of them (81%) are group IB introns. The gene carrying the second more abundant intronic distribution is the *cob* gene with a contribution of 17% of the total examined introns and those introns were identified as subtype ID with a frequency of 45% (fig. 1 and supplementary table 4, Supplementary Material online). The *nad*5 gene (7.5%) with mostly IB introns (48%) and introns of the *rnl* gene (6.5%) mostly typed as IA (28%) follow in abundance. Genes *nad*2, *nad*4, and *nad*4L contain IC introns exclusively, but their allocation is <2% when the total distribution is taken into account (fig. 1 and supplementary table 4, Supplementary Material online). Moreover, IC introns are found mostly in Pezizomycotina (34–87%), with the only exceptions detected in a few cases of Basidiomycota (2–5%) and Saccharomycetes (3–8%). IC introns were not found in the mtDNA of EDF (supplementary table 4, Supplementary Material online). Finally, genes *cox*1, *rnl*, and *cox*2 show the most diverse introns with HEGs variability (at least four different intron types—supplementary table 4, Supplementary Material online). In the majority of the cases, HEGs were found in the P1–P10 or P9 loop, which do not interfere with the intron’s function (supplementary tables 2 and 3, Supplementary Material online).

### Distribution of HEGs in Fungi and mt Genes

In this study, 144 and 413 GIY and LD genes were detected belonging to 40 and 163 species from 30 and 40 different orders, respectively. GIY genes were absent from mt genomes of species belonging to Taphrinomycotina and Cryptomycota. LD genes were found in all examined fungal phyla (table 1).

**Fig. 2.**
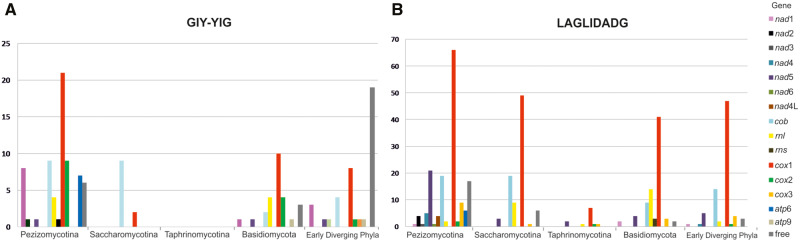
(*A*) GIY-YIG occurrence (quantified in the *y* axis) per gene for all major fungal phyla. Different mt genes carrying introns hosting GIYs are shown in different colored blocks. (*B*) LAGLIDADG occurrence (quantified in the *y* axis) per gene for all major fungal phyla. Different mt genes carrying introns hosting LDs are shown in different colored blocks.

Species from all subphyla contained free GIY and LD genes (few exceptions in phyla of EDF for LD genes, but probably due to the few mt genomes analyzed). The free-standing GIY and LD genes represent the 20% and 7% of the total, respectively (fig. 2 and supplementary table 5, Supplementary Material online). Interestingly, free GIY genes were abundant (57%—fig. 2 and supplementary table 5, Supplementary Material online) in all EDF (with the exception of Zoopagomycota), whereas free LD genes do not reach this level of representation in EDF (5%—fig. 2 and supplementary table 5, Supplementary Material online). Both HEGs were most commonly found in *cox*1 and *cob* genes (fig. 2 and supplementary table 5, Supplementary Material online). Most GIY genes found in Saccharomycetes (73%—8 out of 11 examined) were located at the first intron of *cob* in frame with the preceding exon (supplementary table 2, Supplementary Material online). On the contrary, GIY and LD genes were absent from *nad*4, *nad*4L and *atp*8, *atp*9 genes, respectively.

### Distribution of HEGs per Intron Type

The total characterized introns with GIY and LD ORFs are 107 and 337, respectively. Less than 10% of the examined introns failed to be characterized due to their short size and/or their degenerate sequence (supplementary tables 2 and 4, Supplementary Material online).

**Fig. 3.**
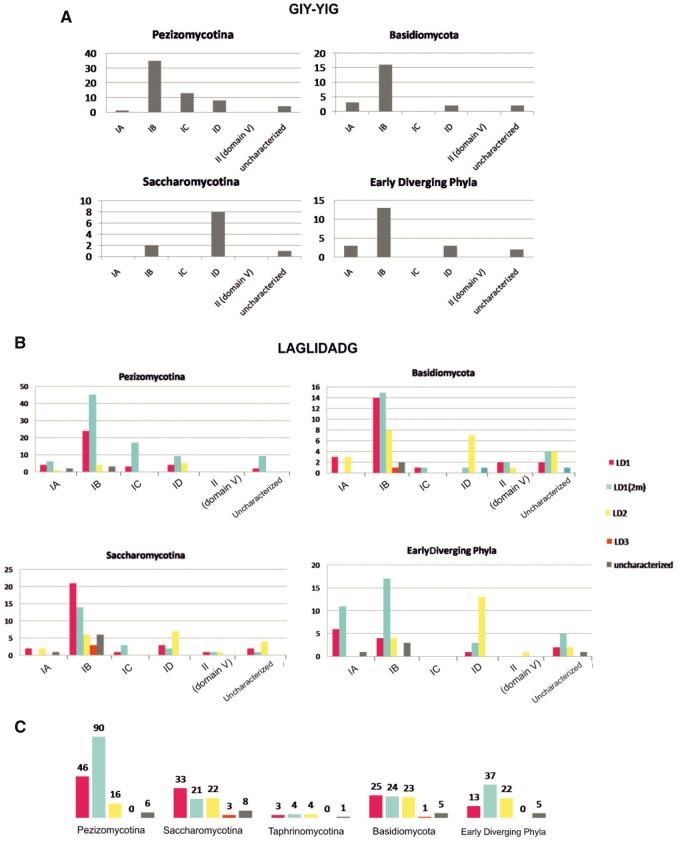
(*A*) GIY-YIG occurrence (quantified in the *y* axis) per intron type for all major fungal phyla. (*B*) LD occurrence (quantified in the *y* axis) per intron type for all major fungal phyla. Different LD types are shown in different colored blocks. (*C*) LAGLIDADG occurrence (quantified in the *y* axis) per fungal phyla. Different LD types are shown in different colored blocks.

Most HEGs were located in IB introns followed by ID introns in all fungal phyla. Subtypes IA and IC carrying HEGs are less commonly found. Subtype IC as intron host was absent from EDF species. For GIY genes, IC host introns were only observed in the Pezizomycotina subphylum and for LD genes only in Ascomycota and Basidiomycota (supplementary table 4, Supplementary Material online and fig. 3*A*).

Group II introns hosted only LD ORFs and only in *cox*1 and in rRNA genes. Group II introns hosting HEGs were totally absent from the examined mt genomes of species belonging to Pezizomycotina (fig. 3*B*).

### Distribution of LDs According to Their Type

The distribution of each LD motif in the intron subtypes was further analyzed. Forty-three percent of the total genes of LD endonucleases is represented by LD1(2m), followed by LD1 (29%), LD2 (20%), and LD3 (2%) (fig. 3*C* and supplementary table 6, Supplementary Material online). Only 7% of the examined LDs remained uncharacterized.

Intronic ORFs encoding LD1 endonucleases, of either one or two motifs, were highly frequent in *cox*1 (160 out of 194 cases, i.e., 82%). Conversely, almost one-third of the LD ORFs in *cob* gene belong to the LD2 type (21 out of 56 cases, i.e., 38%). Genes *nad*4 and *atp*6 have only LD1(2m) in their introns. In all fungal subphyla but Pezizomycotina, ID introns tend to host LD2 endonucleases (supplementary table 6, Supplementary Material online). On the other hand, IB introns in all groups are occupied by LD1 or LD1(2m) genes. Eighty-one percent of IC subtypes host LD1(2m) (supplementary table 6, Supplementary Material online). This distribution reveals the tendency that LD1(2m) have a wider variety of intron target sequences and in extent target genes than the respective LD1 (supplementary table 6, Supplementary Material online).

### Variability of Topology for Free HEGs

Free-standing GIY genes were found in EDF and Pezizomycotina (fig. 2*A*), and free-standing LDs were located in all subphyla with the exception of Taphrinomycotina (fig. 2*B*). In mt genomes of species belonging to EDF, there are four cases (*Rhizophagus intraradices*, *Rhizophagus irregularis*, *Absidia glauca*, and *Rhizophydium* sp.) where the free-standing GIY gene seems to be duplicated, because there are two neighboring copies of the gene within the same genome. The copies have different sizes, but the duplication is supported by the significant identity percentage of those, which is between 31% and 60% and their neighboring topology (supplementary table 2, Supplementary Material online).

Similarly, there are two duplication cases of free LDs, both found in species of “higher” fungi, that is, *Pyronema omphalodes* (Ascomycota) and *Phlebia radiata* (Basidiomycota). In both cases, partial duplications of the neighboring genes *atp*6 and *cox*1 have also occurred in each case, creating new respective pseudogenes (supplementary table 3, Supplementary Material online). The identity percentage was exceptionally high (90% and 100%) when compared with the respective values for GIY duplications.

The genes surrounding the 19 free-standing GIY endonucleases in EDF were identified. Overall, in eight cases, free GIYs were related to *atp*9 (either in proximity, or overlapped, or split to *atp*9a and *atp*9b, or duplicated along with the N-terminus of *atp*9). Seven other GIY genes were located in the proximal region of the *rns* gene. In Pezizomycotina and Basidiomycotina, two cases were found, in which the free GIY of the mtDNA of *P. omphalodes* and *Microbotryum lychnidis-dioicae* were spotted nearby the *atp*9 gene and it seems likely that those cases constitute evolutionary relics of the *atp*9-GIY-YIG batch (supplementary table 2, Supplementary Material online).

In the respective search for the neighboring genes of the free LDs, EDF showed two cases (out of the three) in which *rns* is the neighbor. In Saccharomycetes, free LD2 genes are proximal to *atp*9 gene, which is a hotspot for attracting HEGs and thus produce regional duplications through recombinational events (supplementary table 3, Supplementary Material online).

### Phylogeny: Relationships of HEs

**Fig. 4.**
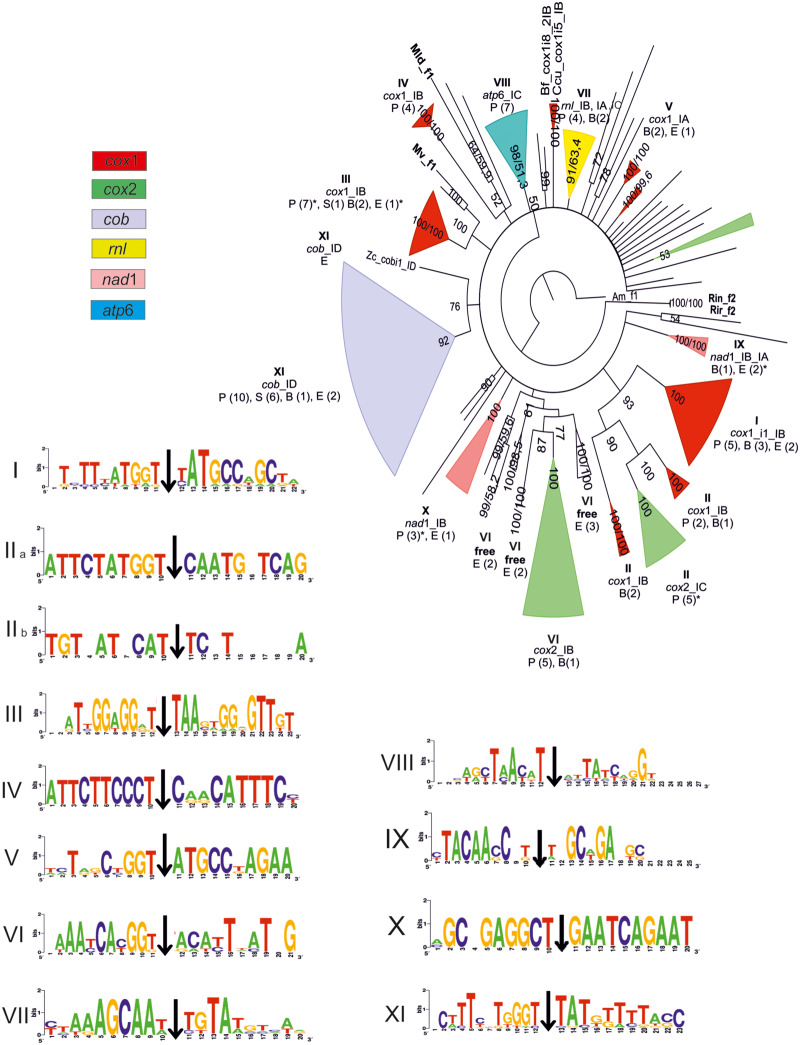
Phylogenetic tree of the GIY-YIG amino acid matrix as produced by employing the BI method. Major clades are shown as filled colored triangles and their different colors indicate different mt genes carrying the introns which hosted the GIYs examined. In detail: mt genes *cox*1, *cox*2, *cob*, *rnl*, *nad*1, and *atp*6 are presented in red, green, gray, yellow, pink, and blue, respectively. Roman numbers show the major clades analyzed in the text and their target insertion sequence of their introns shown additionally as logos. Numbers at the nodes of the tree present the posterior probability (first or unique number) and the NJ-bootstrap (second number, when NJ topology is identical with the respective of the BI tree). Species names are omitted unless they are basal to a cluster discussed in the text (they are provided in supplementary fig. S1, Supplementary Material online). Single letters represent taxonomical units as follows: P, Pezizomycotina; S, Saccharomycetes; B, Basidiomycota; and E, EDF. The parentheses following the single letters represent the number of species found in the examined clusters. Asterisks indicate single alternative topology (either intron subtype or gene or both) among its cluster (see details in supplementary fig. S1, Supplementary Material online).

**Fig. 5.**
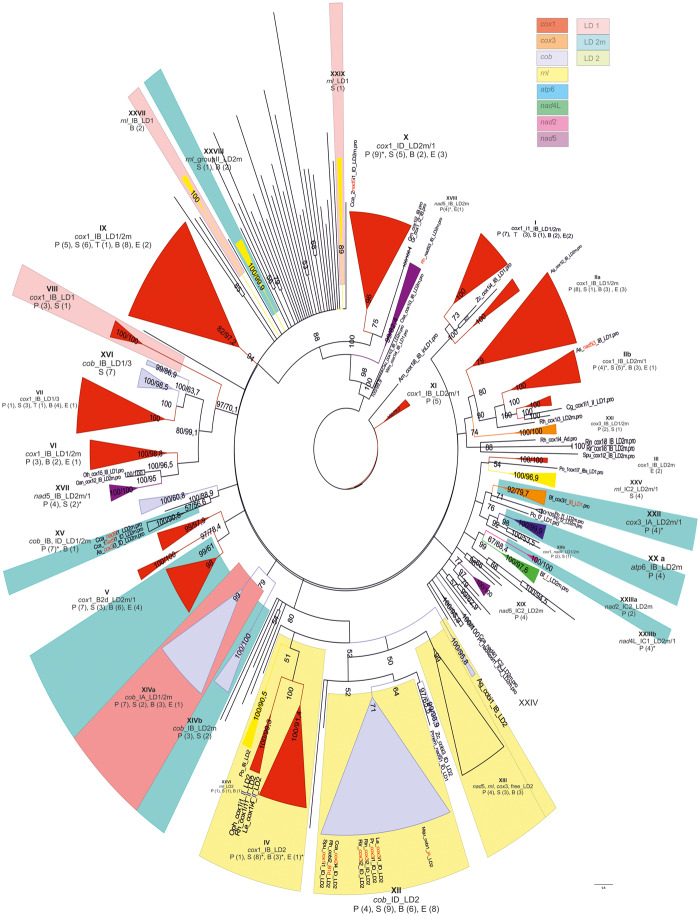
Phylogenetic tree of LAGLIDADG amino acid matrix as produced by employing the BI method. Major clades are shown as filled colored triangles. In detail: mt genes *cox*1, *cox*3, *cob*, *rnl*, *nad*2, *nad*4L, *nad*5, and *atp*6 are presented in red, orange, gray, yellow, pink, green, purple, and blue filled colored triangles, respectively. Highlighted clades with pink, yellow, and blue colors include LD1, LD2, and LD1(2m) subtypes, respectively. Roman numbers show the major clades analyzed in the text. Numbers at the nodes of the tree present the posterior probability (first or unique number) and the NJ-bootstrap (second number, when topology of NJ identical with the respective of the BI tree). Species names are omitted unless they are basal to a cluster discussed in the text (they are provided in supplementary fig. S2, Supplementary Material online). Single letters represent taxonomical units as follows: P, Pezizomycotina; S, Saccharomycetes; T, Taphrinomycotina; B, Basidiomycota; and E, EDF. The parentheses following the single letters represent the number of species found in the examined clusters. Asterisks indicate single alternative topology (either intron subtype or gene or both) among its cluster (see details in supplementary fig. S2, Supplementary Material online).

The Bayesian-based independent phylogenetic trees of 129 GIYs and 339 LDs showed several important clades which determine the evolution of the HEGs. In detail, the strongly supported (>87% PP) clades of the trees contain endonucleases within the same gene and the same intron subtype with a few exceptions (figs. 4 and 5 and supplementary figs. 1 and 2, Supplementary Material online). In order to reveal the phylogenetic relationships of HEGs and their host introns and genes, two factors were taken into account: the subtype of the introns carrying the HEGs and the insertion sites of the introns in the mt genes.

### Phylogeny: Relationships of GIY-YIG

The root of the tree is a mt free-standing GIY of *A. macrogynus*. Other free GIYs of EDFs are also located basally. Each free-standing GIY gene is at the base of a cluster with intronic GIY ORFs usually located in introns of the same mt gene (fig. 4 and supplementary fig. 1, Supplementary Material online). This is an indication that the insertion of free GIYs into the introns of mt genes have happened several different independent times in the evolution of the mt genomes.

GIYs placed within introns of the same subtype of a mt gene, cluster together. For instance, clades VIII and XI include GIY sequences located in IC and ID introns of the *atp*6 and *cob* genes, respectively (fig. 4). In the case of *cob*, ORFs from all phyla are predominantly found in the first intron of this gene, fused in frame with the preceding exon with a significant conservation in their target sequences.

There is a single case where the mt gene, that is, *rnl*, has all GIYs in a single clade, irrelevant to the intronic subgroup that hosts these HEGs (fig. 4, Clade VII). However, the conservation of the insertion sequence of the introns shows that this domain of the gene is a hotspot of introns carrying GIY genes irrelevant to the intron’s subtype (fig. 4, Clade VII and its logo).

HEs from *nad*1 introns are grouped in two separate clades. The one is formed by representatives of EDF and Basidiomycota and shows variable intronic insertional sites (fig. 4, Clade IX and its logo). The second clade includes GIYs from one EDF and three Pezizomycotina species with high conservation in their target sequences (fig. 4, Clade X, Logo X).

The insertion of GIY genes in introns of *cox*1 seems to have occurred many independent times through mt genome evolution. At the base of the four major *cox*1 clades, a free-standing GIY from an EDF’s genome is located (fig. 4, Clades I–IV). The insertion sequence from each clade indicates high conservation (fig. 4, Logos I–IV). Clade I consists of GIYs placed in the first intron of the *cox*1 gene. Respective endonucleases from other IB introns within *cox*1 showed a close relation to Clade I (with bootstrap support >90%), but they were distinct (Clade II). Clades III and IV include representatives in IB introns of *cox*1 from all fungal phyla (fig. 4, Clades III and IV). Three GIYs found in introns of *cox*1 from two Basidiomycota and an EDF species (i.e., *Lentinula edodes*, *P. radiata*, and *Zancudomyces culisetae*—supplementary fig. 1, Supplementary Material online) form Clade V with the novelty of being allocated to introns of subgroup IA.

GIYs encoded by ORFs in introns of *cox*2 compose two district clades. Clade II representatives are positioned in IC introns and are related to intronic HEs in IB introns of *cox*1 (fig. 4, Clades I and II). On the contrary, Clade VI seems to have originated from GIYs of ancestral free-standing HEs similar to modern genes found in EDF (fig. 4, Clade VI).

The results found for the phylogeny of GIY endonucleases overall also apply to the HEG phylogeny within the different phyla of EDF, even though they are underrepresented (supplementary fig. 3, Supplementary Material online).

### Phylogeny: Relationships of LAGLIDADG

Similarly to the GIY phylogeny, the major clades are composed by LDs within introns of the same subgroup. For instance, LDs in ID introns of *cox*1 (fig. 5, Clade X) are grouped separately from LDs of IB introns (fig. 5, Clade IX) but in a few cases, they remain as sister clades. A typical example is the formation of the sister clades IVa and XIVb in *cob*, which consist of LDs found in IA and IB introns, respectively. In accordance with the GIY phylogeny, based on their scattered distribution, the insertion of LD genes in introns of *cox*1 resulted in multiple independent events (fig. 5, clades highlighted in red). These results also apply to the HEG phylogeny within the different phyla of EDF (supplementary fig. 4, Supplementary Material online).

LD endonucleases of the same type tend to group together. Clades from HEs found at *atp*6 (fig. 5, Clade XXa) and *nad*2 genes (fig. 5, Clade XXIIIa) include LD1(2m), whereas Clades VIII of *cox*1 and XXVII of *rnl* have one motif LD1 exclusively. On the other hand, within clades of *cox*3 (fig. 5, Clades XXI and XXII) and *cox*1 (e.g., fig. 5, Clades I and X), there are LDs of either one (LD1) or two motifs LD1(2m). Conclusively, the one and two motifs of LD1 show a mixed distribution in different clades.

**Fig. 6.**
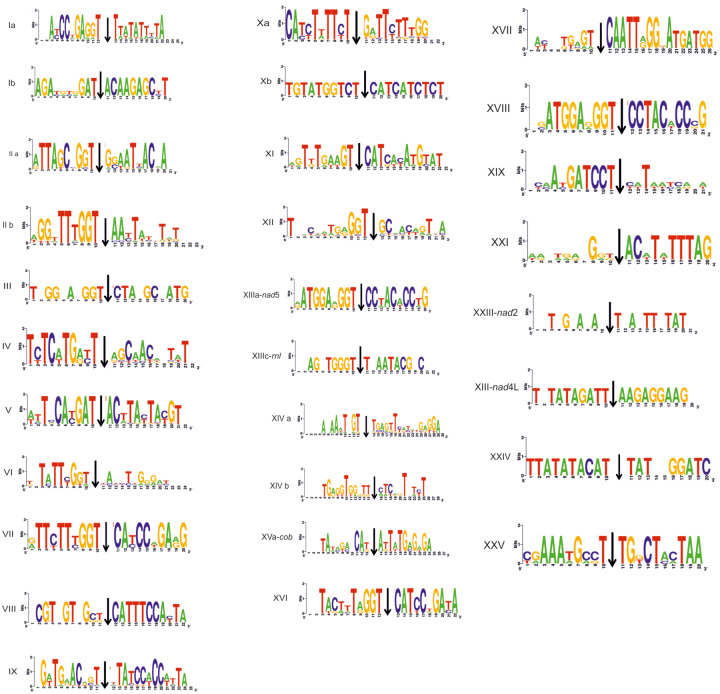
Logos of the target insertion sequence of introns carrying LAGLIDADG. Roman numbers correspond to the respectively numbered major clades of the tree shown in figure 5.

LD2 endonucleases seem to separate from LD1 (fig. 5 and supplementary figs. 2 and 4, Supplementary Material online). In some clades, LD2 sequences from different genes but same intron types are grouped together. For instance, Clade XII includes LD2 sequences in ID introns mainly from *cob* and *cox*3, and exceptionally from *cox*1 and *cox*2 genes (fig. 5). The basal taxon of this clade refers to an LD2 in the *cob* gene of *Zancudomyces culisetae*, which most probably resembles to the ancestral form. The target sequence of the intron is significantly conserved, especially in proximity to the intron’s insertion site (range of 6 bp) (fig. 6). LD2 in group II introns of EDF such as *Rhizophydium* are grouped with those in group IB introns of *cox*1 gene of Pezizomycotina, Basidiomycota, and Saccharomycetes (fig. 5, Clade IV). The 3′ end of the exon preceding the target sequence of the intron and the first 6 bp of the 5′ end of the following exon are almost identical in all cases (fig. 6).

In this phylogenetic work, LD3 are grouped with one motif LD1 (or LD that could not be characterized using BlastP). This shows a phylogenetic relationship between LD3 and LD1 endonucleases. The intron insertion sequences even in the case of LD3 remain conserved and identical to insertion sites of LD1 (fig. 6).

### Horizontal Gene Transfer

Horizontal gene transfer (HGT) events were examined using LD and GIY protein sequences from the mtDNA of representative phytopathogenic (*Microbotryum lychnidis-dioicae*, *Ustilago maydis*, *Fusarium oxyporum*, and *Phaeosphaeria nodorum*), entomopathogenic (*Cordyceps bassiana*, *Candida corydali*, and *Cyberlindera suaveolens*), saprophytic (*P. radiata* and *A. macrogynus*), and symbiotic species like lichens (*Peltigera malacea* and *Peltigera membranecea*).

In GIY analyses, five HGT events among HEGs found in introns of two mt genes from phytopathogenic, entomopathogenic, and lichenized species to species belonging to α-proteobacteria, firmicutes, and actinobacteria were found (supplementary table 2, Supplementary Material online). These HGT phenomena were supported by the high percentage of homology (range: 61–87% identity and 74–93% positives). There were four HGT events (range: 73–97% identity and 83–97% positives) among LD genes found in introns of three mt genes from phytopathogenic species to another phytopathogenic fungus and α-, β-, and γ-proteobacteria (supplementary table 3, Supplementary Material online).

## Discussion

In the 1.45 BYA history of the mt genome evolution (Martin and Mentel 2010), the mt genome has undergone significant reduction in genome complexity and size through the loss of protein-coding genes, intronic sequences, and intergenic regions (Gray et al. 1999; Adams and Palmer 2003; Pogoda et al. 2019). However, fungal mt genomes present significant size variability which is partly attributed to the abundance of introns (Kouvelis et al. 2004; Hausner 2012; Jalalzadeh et al. 2015). This variability is further extended with the inclusion of HEGs from the families of GIY-YIG and LAGLIDADG in the introns (Lambowitz and Belfort 1993; Lang et al. 2007).

### Intron Evolution

Ancestral bacterial introns were common, according to the “intron-early” theory (Koonin 2006; Wang et al. 2016) and rapidly proliferated to multiple genomic sites after their endosymbiotic transformation to mitochondrion (López-García and Moreira 2006; Koonin 2016). According to this theory and the study of group I introns found in *cox*1 in eukaryotes (Férandon et al. 2010), the trend of mt genome evolution was toward the loss of introns. However, our analysis showed that this was not always the case. Some introns might have remained intact at conserved gene locations, showing their ancestral origin, like the group IA intron found in *omega* site of the *rnl* gene (Korovesi et al. 2018; Wai et al. 2019) or the group ID intron (carrying a GIY-YIG gene in frame with the upstream exon) located in *cob* (this study). This domestication of the ancestral introns showed an adaptation to their host gene which may be explained as a result of lacking mobility (Novikova and Belfort 2017), or of their pertinent role to the stability of the gene that hosts the intron (Schafer 2003; Korovesi et al. 2018). Other introns proved to be acquired only lately in evolution, either through HGT events or through active transposition (Mardanov et al. 2014; Wu et al. 2015). The transposition of these introns to other genomic regions with less sequence similarity was spread further under stress-induced conditions, as other studies have experimentally suggested (Coros et al. 2009; Robbins et al. 2011). A recent study about introns in nuclear genes of *Saccharomyces cerevisiae* also showed that they play crucial role in the survival of the organism under starvation conditions (Parenteau et al. 2019).

### Intron–HEGs Coevolution

The representation of mt HEGs within introns and their phylogenetic relationships found in species from all fungal phyla are shown in this work. They support the idea that introns, with the inclusion of HEGs, may provide an advantage to the survival of the organism. The localization of HEGs in peripheral loops of the introns limited the cost of their intervention and played crucial role in *cis* splicing. These composite introns were leaping in other genes with similar target sequences in a *cis* mode of action, even though the ability of HEs to act also in *trans* cannot be excluded (Nadimi et al. 2012). The basal placement of free-standing HEs to the phylogenetic trees of this work, pinpoint their ancestry and the possible change of their role from sole mobile elements to an intron homing status. This argument is further supported in this study, because it was found that free-standing HEGs are mostly a common characteristic of the “primitive” species, that is, of Blastocladiomycota, Chytridiomycota, and Mucoromycota. It has been shown in previous studies that HEGs were mobile elements independent of a host intron (Sellem and Belcour 1997; Edgell et al. 2011), but by targeting the same sequences, introns and HEGs were united afterward to create the composite mobile elements (Bonocora and Shub 2009; Zeng et al. 2009), which can be found nowadays. Therefore, the coevolution of intron and HEGs is anticipated and HEGs are necessary particles of their introns.

### Mechanisms of Coevolution

**Fig. 7.**
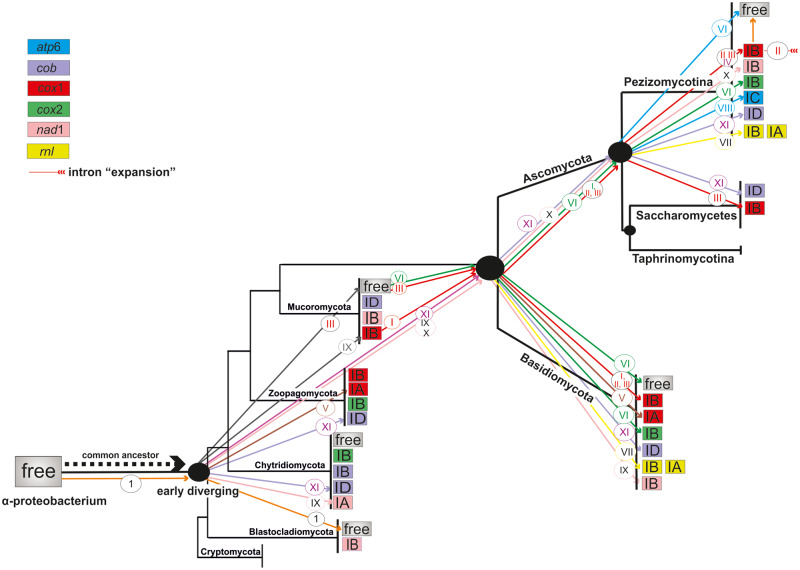
Suggested model for the evolution of GIY-YIG endonucleases in a dendrogram showing the main taxonomical fungal groups (phyla of fungi and subphyla of Ascomycota) according to the phylogeny shown in Ahrendt et al. (2018). Different colors of the boxes correspond to different host genes (red: *cox*1, green: *cox*2, light purple: *cob*, yellow: *rnl*, light pink: *nad*1, blue: *atp*6, and gray: free-standing ORFs) and inside the boxes host intron subtypes are demonstrated. Colored arrows and roman numbers within circles above the arrows correspond to the phylogenetic clusters shown on the respective phylogenetic tree (fig. 4). Arrows with number 1 in a circle denotes the independent mobility of the free GIY gene.

**Fig. 8.**
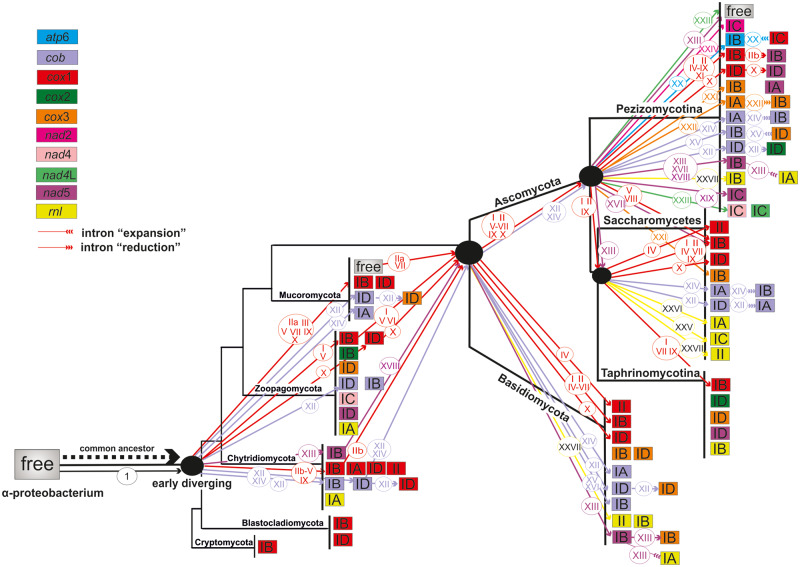
Suggested model for the evolution of LD endonucleases in the fungal kingdom in a dendrogram showing the main taxonomical fungal groups (phyla of fungi and subphyla of Ascomycota) according to the phylogeny shown in Ahrendt et al. (2018). Different colors of the boxes correspond to different host genes (red: *cox*1, green: *cox*2, orange: *cox*3, light purple: *cob*, yellow: *rnl*, pink: *nad*2, light pink: *nad*4, light green: *nad*4L, purple: *nad*5, blue: *atp*6, and gray: free-standing ORFs) and inside the boxes host intron subtypes are demonstrated. Colored arrows and numbers within circles above the arrows correspond to the phylogenetic clusters shown on the respective phylogenetic tree (fig. 5). Arrows with number 1 in a circle denotes the independent mobility of the free LD gene.

Introns commonly found in all phyla can be derivatives from ancestral states and lately acquired composite elements may be found only in mt genes of Pezizomycotina (Edgell et al. 2011; Deng et al. 2018; Zubaer et al. 2018). Our analyses, not only supports this theory, but for the first time introduces the notion that this invasion of the ancestral HEGs had introns of subgroup IB as preferential targets and secondarily, those of ID and IA (figs. 7 and 8). Based on the above arguments, composite IB and ID introns in *cox*1 and *cob* as well as IA introns in *cob* may be considered ancestral elements of the fungal mitogenomes. In contrast, IC introns found in *atp*, *nad*, and rRNA genes acquired ORFs more recently (figs. 7 and 8). Secondary structures of the group I subtypes have shown that group IB introns are the most compact forms (with the smallest number of conserved helices), followed by subgroups IA and ID (Cech et al. 1994; supplementary table 7, Supplementary Material online). The most recently found introns, that is, of subgroup IC, contain nine more conserved helices in comparison to group IB introns (supplementary table 7, Supplementary Material online). It indicates the size expansion of introns with the addition of new helices due to the insertion of the HEGs. This intron’s expansion is in contradiction to the widely accepted theory that the mt genomes were shrinking in size through evolution (Lang et al. 1999).

The recognition of similar sequences from the HEs motivated the transposition of composite elements in a variety of targets. The “GGT” motif before the 5′ end splicing site is conserved throughout, as shown from all logos created (fig. 6). The 5′ primed sequence tends to be crucial to the transposition mechanism of the introns carrying LD endonucleases. The 3′ primed sequence shows a tendency of being a “CAT” motif (fig. 6). In the case of introns hosting a GIY, these motifs exist but with considerable exceptions (fig. 4). Until now, only the importance of the 3′ primed target sequences has been verified as potential recombination hotspots from free-standing endonucleases next to intronless mt genes in yeasts (Wu and Hao 2019).

### Proposed Models of GIY and LD Evolution

After the comparative analyses of all HEGs, GIY genes were preferably found in fusion with their upstream exon. This tendency was previously found in the *cob* gene (Guha et al. 2018). It was suggested that this fusion provides the endonucleases with access to the *cis* genetic elements that are required for their expression (Guha et al. 2018) after a proteolytically maturation process (Pellenz et al. 2002). From this analysis, it becomes evident that this stability in the structure of the mt genome contributes to the elusion of recombination which may have further shuffled the mt gene content by creating new pseudogenes, as it has happened in the genomic region of *atp*9 (Kolesnikova et al. 2019; this study).

The analyses of the LDs showed that these HEGs had more variable target sequences compared with the respective sequences of GIY. Our results indicate a distinct (probable ancestral) origin of LD2 endonucleases, because LD2 endonucleases are grouped separately from LD1 and there was a tendency to switch from LD2 endonucleases to LD1 (or LD1(2m)) during the fungal evolution. The one and two motifs of LD1 show a scattered distribution in the different tree clades, which suggests that those two LD versions originate from each other, by either duplication of the one motif or loss of the second motif. LD1 gave rise to LD3 in the lineage of Saccharomycetes (and in a single case of a species from Basidiomycetes) by accumulating mutations and changing their protein sequence. LD2 seem to be transposed between same intron types of different genes, whereas LD1 and LD1(2m) intermingle with no clear distribution (fig. 5). Additionally, LD1(2m) presented the most significant variability. The higher percentage of LD(2m) endonucleases indicates a dominance of this type of endonuclease throughout the evolution against the other LD types. Their supremacy was further confirmed because LD1(2m) endonucleases present a variety of target genes and intron types and thus, mobilize to novel targets such as IC introns. Their ability to recognize nonsymmetrical target sequences has been previously investigated (Chevalier et al. 2005). The LD1(2m) act as reactive monomers, in contrast to LD1 which are dimerized in order to be active (Lucas et al. 2001). Another advantage for the domination of LD1(2m) is that intronic LD1(2m) renders its host more independent as far as its splicing and transposition is concerned. Introns’ mobility dependence was experimentally confirmed in the *cox*1 intron of *Schizosaccharomyces pombe* (Pellenz et al. 2002). After all, introns carrying these endonucleases may act in a selfish way, and in this way HEGs are able to reproduce and be transposed into new sites, simultaneously with their hosts as composite elements. In this manner, LD1(2m) secures its own position and in extent, their evolution. A theory which is further supported by the fact that LD1 is the majority and mostly found in lately evolved species like Pezizomycotina, whereas the early EDF contain often the “archaic” LD2 (see Results).

Group II introns carrying LDs were scarce and only in *cox*1, *rnl*, and *rns* genes of mt genomes from all fungal subphyla, whereas there were no group II introns hosting GIY genes. This is the result of the LD mobility with a mechanism similar to the intronless homing of HEGs as proposed for group I introns (Hausner et al. 2014). This patchy distribution may be attributed to multiple independent HGT phenomena of LDs. Moreover, the basal phylogenetic positioning of LD2 in group II introns of EDF (i.e., *Rhizophydium*) to LD2 in group IB introns of *cox*1 gene of Pezizomycotina, Basidiomycota, and Saccharomycetes (fig. 5, Clade IV) was observed. This indicates the transposition of the LD2 gene from the group II introns to group IB introns in later (and probably multiple) evolutionary events.

### The “Aenaon” Model

**Fig. 9.**
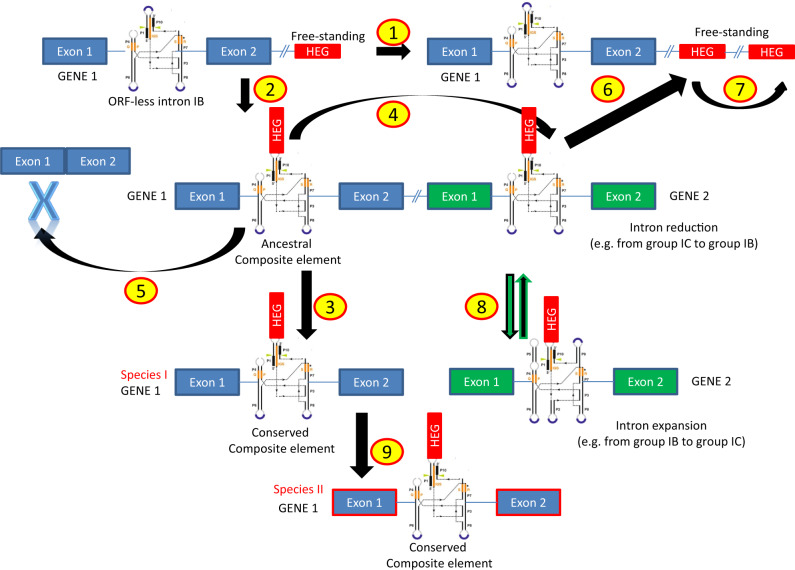
Schematic presentation of the main events of the “aenaon” model. “HEG” in red rectangle represents the gene of GIY or LD endonuclease. Numbers in circles describe the steps/mechanisms as follows: (1) Independent vertical gene transfer of introns and HEGs, (2) vertical gene transfer and creation of composite element (“homing”), (3) vertical gene transfer of composite elements, (4) transposition of composite elements to new loci (i.e., other mt genes), (5) elimination of introns and HEGs, (6) mobility of HEG into new loci (outside of mt genes), (7) duplication of free-standing HEG within the mt genome (with probable additional creation of pseudogenes—e.g., cases of *atp*9), (8) genetic recombination and other duplication events either “expanding” (e.g., IB to IC) or “reducing” intron structures (e.g., IC to IB), and (9) HGT event of the composite element from a mt genome of species I to the respective genome of species II.

The evolution of HEGs and their intronic hosts seems to follow many dynamic steps. The mt genome variability in the fungal mt genomes throughout the evolution may be explained with a new model, the “aenaon” (meaning restless/perpetual) model (fig. 9). This model combines characteristics of the previously “debating” two models, that is, the “intron-late” and the “intron-early” theories (Koonin 2006). In brief, based on the literature and these results, the main arguments for the “aenaon” model are 1) there are ancestral introns and HEGs which throughout the fungal evolution are located within the same locus and have conserved site recognition, 2) mt introns evolved toward two directions: mobility, that is, similar target sites but different actual locations (e.g., *cox*1 *and cox*2) and “expansion” that is, their actual structure evolved from the ancestral compact form to a modern “expanded” one with new added hairpins (e.g., from IB toward IA or IC—see figs. 7–9), 3) the reverse “reduction” is less common but still exists (e.g., from IA to IB—see figs. 8 and 9), and 4) the ancestral introns showed a bias to be extinct as the endosymbiotic model indicates a tendency for shrinking the mt genome size. The assembly of the mitochondrion is achieved nowadays with the participation of the nucleus, after the transfer of a-proteobacterium’s preexisting genes to the nucleus in one “big” event (Koonin 2006). Thus, the ancestral introns are “living” remnants of this procedure, 5) there are “new” introns usually found in mt genes of “higher” fungi which were acquired recently and provide evidence of the mt genome expansions (Lee et al. 2019) and 6) free-standing HEGs were found at the beginning of evolution but nowadays in “higher” fungi are mostly “intron homing” and structurally stable. HEGs may follow their own evolutionary pathway but usually they are attached to the intron which they invaded. Throughout evolution, mutations have occurred and changed the background and the moving ability of the HEGs. The mechanism of their invasion was the nonhomologous recombination but mutations have diminished this possibility (Brankovics et al. 2018; Guha et al. 2018). Finally, the trend was toward the invasion of HEGs to the introns and less often the other way around, that is, HEGs to become independent. Thus, the “aenaon” hypothesis shows a restless perpetual coevolution of introns and HEGs which contributes to the diversity of fungal mt genomes.

## Supplementary Material

Supplementary data are available at *Genome Biology and Evolution* online.

## Supplementary Material

evaa126_Supplementary_Data
